# Voluntary Exercise Induces Astrocytic Structural Plasticity in the Globus Pallidus

**DOI:** 10.3389/fncel.2016.00165

**Published:** 2016-06-21

**Authors:** Kouko Tatsumi, Hiroaki Okuda, Shoko Morita-Takemura, Tatsuhide Tanaka, Ayami Isonishi, Takeaki Shinjo, Yuki Terada, Akio Wanaka

**Affiliations:** ^1^Department of Anatomy and Neuroscience, Faculty of Medicine, Nara Medical UniversityKashihara, Japan; ^2^Department of Functional Anatomy, Graduate School of Medical Sciences, Kanazawa UniversityKanazawa, Japan; ^3^Department of Anesthesiology, Faculty of Medicine, Nara Medical UniversityKashihara, Japan

**Keywords:** Olig2, astrocyte, voluntary exercise, structural plasticity, globus pallidus

## Abstract

Changes in astrocyte morphology are primarily attributed to the fine processes where intimate connections with neurons form the tripartite synapse and participate in neurotransmission. Recent evidence has shown that neurotransmission induces dynamic synaptic remodeling, suggesting that astrocytic fine processes may adapt their morphologies to the activity in their environment. To illustrate such a neuron-glia relationship in morphological detail, we employed a double transgenic Olig2^CreER/WT^; ROSA26-GAP43-EGFP mice, in which Olig2-lineage cells can be visualized and traced with membrane-targeted GFP. Although Olig2-lineage cells in the adult brain usually become mature oligodendrocytes or oligodendrocyte precursor cells with NG2-proteoglycan expression, we found a population of Olig2-lineage astrocytes with bushy morphology in several brain regions. The globus pallidus (GP) preferentially contains Olig2-lineage astrocytes. Since the GP exerts pivotal motor functions in the indirect pathway of the basal ganglionic circuit, we subjected the double transgenic mice to voluntary wheel running to activate the GP and examined morphological changes of Olig2-lineage astrocytes at both the light and electron microscopic levels. The double transgenic mice were divided into three groups: control group mice were kept in a cage with a locked running wheel for 3 weeks, Runner group were allowed free access to a running wheel for 3 weeks, and the Runner-Rest group took a sedentary 3-week rest after a 3-week running period. GFP immunofluorescence analysis and immunoelectron microscopy revealed that astrocytic fine processes elaborated complex arborization in the Runner mice, and reverted to simple morphology comparable to that of the Control group in the Runner-Rest group. Our results indicated that the fine processes of the Olig2-lineage astrocytes underwent plastic changes that correlated with overall running activities, suggesting that they actively participate in motor functions.

## Introduction

Accumulating evidence suggests that neuron-glia interaction is important in maintenance of the nervous system (Welberg, 2009). Activity-dependent structural changes occur in glial elements as well as neurons (Molofsky et al., 2012). The majority of morphological studies of astrocytes make use of glial fibrillary acidic protein (GFAP) immunoreactivity, because GFAP is an established and widely used marker for mature astrocytes. While some groups have reported that physical exercise decreased GFAP expression in the hippocampus (Bernardi et al., 2013), others have shown that it increased GFAP expression in different brain regions, such as the cerebral cortex, striatum and hippocampus (Li et al., 2005; Rodrigues et al., 2010; Saur et al., 2014). In some cases, no alteration of GFAP expression in response to physical exercise was observed in the rat hippocampus (de Senna et al., 2011). These contradictory results between studies probably arise from methodological differences including animal species or strains, brain regions, exercise protocols and duration of exercise training (Viola and Loss, 2014). All the above studies monitored GFAP; however, GFAP expression is not uniformly detected in astrocytes, and in some brain regions it is totally undetectable (Lee et al., 2006, 2008). As shown by intracellular dye filling (Haber et al., 2006; Halassa et al., 2007) or the brainbow technique (Livet et al., 2007), astrocytes have very complex “bushy” morphologies with lamellipodial processes, and these fine processes are devoid of GFAP (Bushong et al., 2002). Such heterogeneity of intercellular and intracellular GFAP localization may also contribute to the discrepancies regarding the effect of physical exercise on astrocytes.

The transcription factor Olig2 has an essential role in generating oligodendrocytes and motor neurons in the embryonic stage (Takebayashi et al., 2002). In the adult brain, most Olig2-positive cells co-express NG2 proteoglycan and are termed adult oligodendrocyte progenitor cells (OPCs; Goldman, 2003). These cells generate mainly OPCs/NG2 glia or mature oligodendrocytes and a small number of astrocytes, but no neurons, in the adult brain (Dimou et al., 2008). We have been tracing lineages of Olig2 promoter-active cells in the adult mouse forebrain using Olig2^CreER/WT^; ROSA26-GAP43-EGFP mice that express plasma membrane-targeted GFP in physiological and pathological conditions (Tatsumi et al., 2008; Islam et al., 2009; Okuda et al., 2009). The membrane-targeted GFP clearly revealed the detailed morphology of oligodendrocytes (Islam et al., 2009), OPCs/NG2 glia (Okuda et al., 2009) and astrocytes (Tatsumi et al., 2008). During these lineage-tracing studies, we noticed that Olig2-lineage astrocytes localized in restricted regions in the gray matter. These cells preferentially existed in the globus pallidus (GP), which is a component of the indirect pathway of the basal ganglionic circuits. Recent reports have indicated that the direct pathway and the indirect pathway of basal ganglionic circuits coordinate outputs through the thalamic nuclei and determine the activities of the motor cortex (Sano et al., 2013; Calabresi et al., 2014).

Physical exercise enhances the behavioral performance of animals in spatial memory tasks (Burghardt et al., 2004). The beneficial effect of physical exercise may, in part, be attributed to upregulated neurotrophins, which would help to protect neural functions (Hillman et al., 2008). In addition to these effects on neurons, recent studies have demonstrated that physical exercise induces structural and biochemical changes in astrocytes as well as in neurons (de Senna et al., 2011; Bengoetxea et al., 2013). These studies suggested that the Olig2-lineage astrocytes in the GP can be a good model to delineate the relationship between neuronal activities and astrocytic morphologies. In the present study, we subjected the double-transgenic mice to voluntary wheel running to activate the GP and focused on detailed morphological changes of Olig2-lineage astrocytes, taking advantage of the membrane-targeted GFP. We show that the morphology of Olig2-lineage astrocytes, especially of their fine processes, correlates well with running wheel activity at both light microscopic and electron microscopic levels.

## Materials and Methods

### Animals

Olig2 knock-in mice (Olig2^KICreER^; Takebayashi et al., 2002) and ROSA26-GAP43-EGFP reporter line (Nakahira et al., 2006) were kindly provided by Prof. Takebayashi at Niigata University School of Medicine and Prof. Ono at Kyoto Prefectural University of Medicine. Olig2^KICreER/WT^ mice were crossed with a reporter line to obtain Olig2^KICreER/WT^; ROSA26-GAP43-EGFP mice (hereafter termed Olig2-GFP mice). The genetic background of the Olig2-GFP mice was mixed of the C57 black six strain and the 129 X1/svJ strain (Takebayashi et al., 2002; Nakahira et al., 2006). Mice were housed in standard cages under 12-h light/dark cycle and temperature-controlled conditions. Food and water were available *ad libitum*. All mice were male and 12 weeks old, and were housed individually. All the protocols for the animal experiments were approved by the Animal Care Committee of Nara Medical University in accordance with the policies established in the NIH Guide for the Care and Use of Laboratory Animals.

### Experimental Design

**Figure 1:**
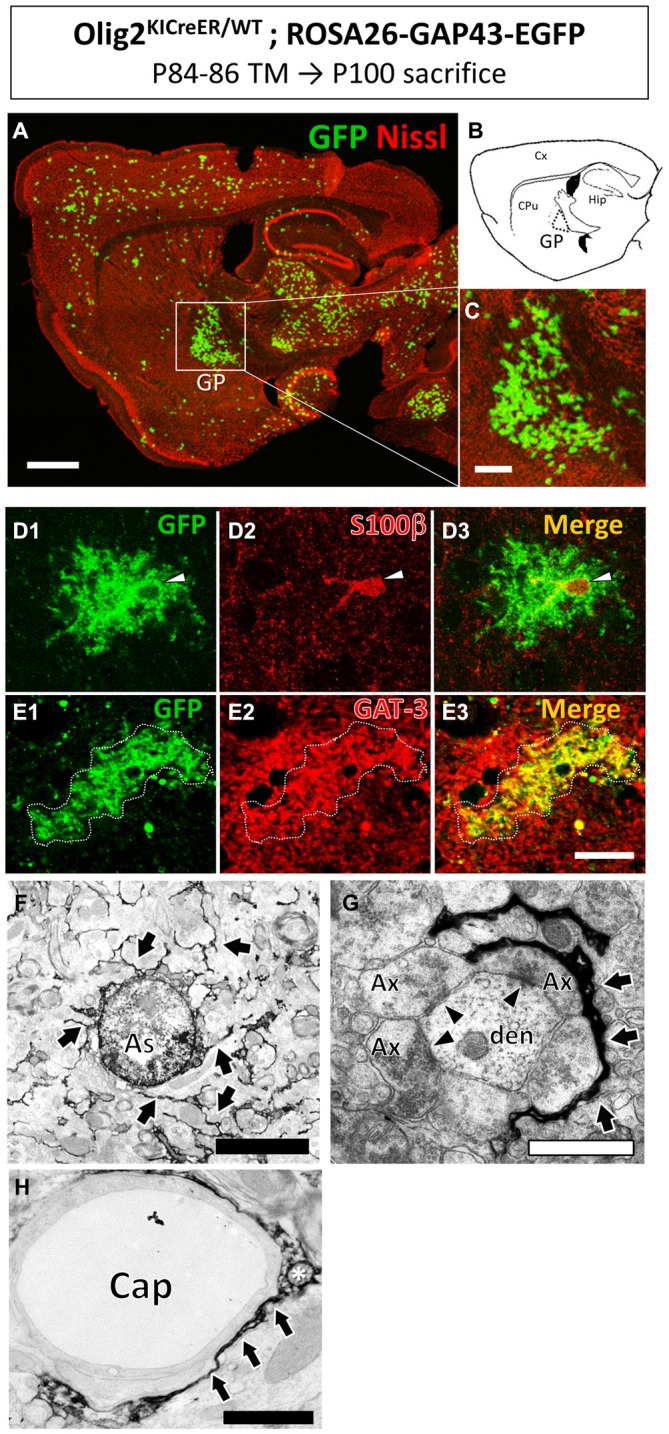
**Olig2-lineage astrocytes localized in the globus pallidus (GP).** Olig2^KICreER/WT^; ROSA26-GAP43-EGFP mouse was treated with TM from postnatal day 84–86 and sacrificed at postnatal day 100. The brain was dissected out and subjected to GFP immunohistochemistry. **(A)** Low-magnification view of GFP-positive Olig2-lineage cells (green) in a sagittal brain section labeled with red fluorescent Nissl stain. **(B)** Schematic representation of the sagittal section corresponding to **(A)** indicates brain regions such as the cerebral cortex (Cx), caudate-putamen (CPu), hippocampus (Hip), and GP. Boxed area in (**A**; GP) is magnified in **(C)**. Olig2-lineage cells with strong GFP-immunoreactivity have bushy morphology and are easily detectable at this low magnification. Note that the bushy cells preferentially cluster in the GP. **(D)** The nucleus of Olig2-lineage bushy-type cell (**D1**; arrowhead) is s100β-positive (**D2**; arrowhead). **(D3)** Shows the merged image of **(D1,D2). (E)** An Olig2-lineage bushy-type cell (**E1**; outlined with dotted line) also expressed GABA transporter (**E2**; GAT-3; red color). **(E3)** Shows merged image of **(E1,E2). (F–H)** Immunoelectron microscopy revealed features of protoplasmic astrocytes at the ultrastructural level. The cellular processes extended into the neuropil **(F**, arrows) and higher-magnification view shows immunoreactive perisynaptic processes wrapping around synapses (**G**, arrows). Arrowheads indicate synapses with postsynaptic density **(G)**. An immunoreactive process attaches to a brain capillary (Cap) as a form of endfoot (**H**, arrows). The asterisk shows a mitochondrion in the astrocytic process **(H)**. Ax, axon; den, dendrite. Scale bars: **(A)** 1 mm; **(C)** 500 μm; **(E3)** (also for D1–3 and E1,2) 20 μm; **(F)** 5 μm; **(G,H)** 1 μm.

**Figure 2:**
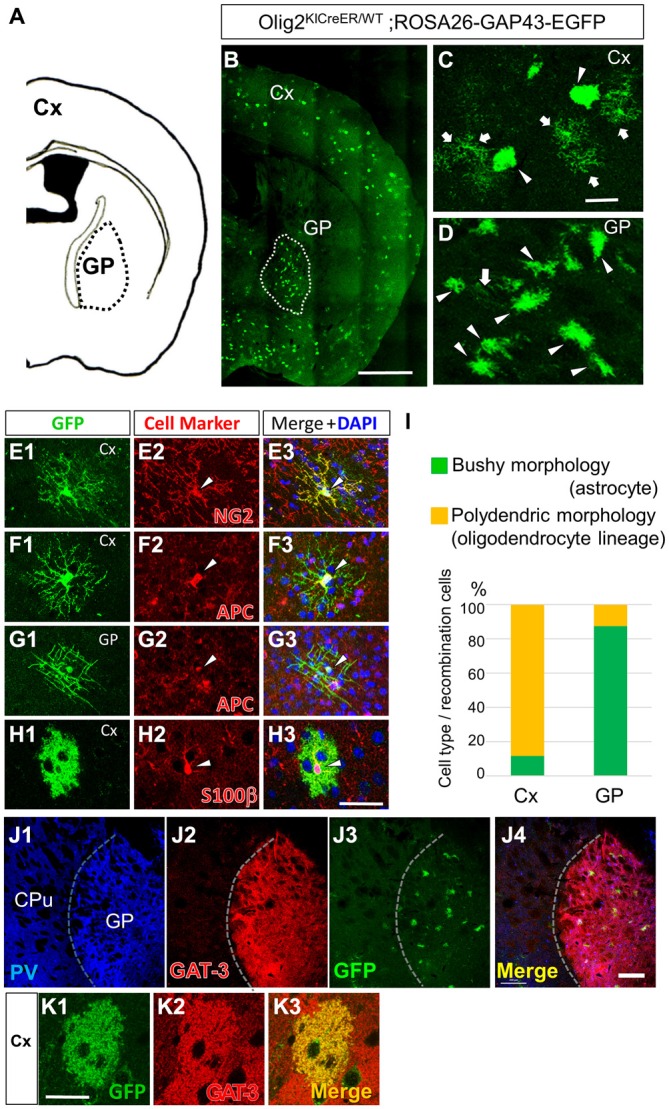
**Olig2-lineage astrocytes are relatively abundant in the GP. (A)** Diagram showing the GP and cortex (Cx) of a mouse coronal section (0.46 mm posterior to the bregma). **(B)** Low-magnification view of GFP-positive Olig2-lineage cells in Olig2^KICreER/WT^; ROSA26-GAP43-EGFP mice (the section is at the same level as that in **A**). Olig2-lineage cells preferentially cluster in the GP (demarcated with a dotted white line). **(C,D)** Higher-magnification views of Olig2-lineage cells in the Cx **(C)** and the GP **(D)**. Arrows indicate cells with polydendric morphology and arrowheads indicate cells with bushy morphology. Note that bushy-type cells predominate in the GP, while polydendric-type cells do so in the Cx (see also **I**). **(E–H)** Double immunofluorescence with cell marker antibodies shows that polydendric-type cells in the Cx are NG2 proteoglycan-positive (**E2**; arrowhead, OPC/NG2 glia) or APC-positive (**F2**; arrowhead, oligodendrocyte). The polydendric-type cells in the GP are also positive for APC (**G2**; arrowhead, oligodendrocyte) and bushy-type cells in the cortex are S100β-positive (**H2**; arrowhead, mature astrocyte). Scale bars: **(B)** 1 mm; **(C)** 30 μm (also for **D**); **(H)** 30 μm (also for **E–G**). **(I)** Quantitative analyses of Olig2-lineage cells in the Cx and the GP revealed that 88% of cells are polydendric-type (OPCs/NG2 glia or mature oligodendrocytes) in the Cx, while 87% are bushy-type (mature astrocytes) in the GP. **(J)** Lower-magnification views of a triple-immunolabeled section. The GP had many PV-positive (GABAergic) neurons **(J1)**, while the neighboring striatum showed fewer. The border between the two regions is indicated by a dotted line. The GP also expressed GAT-3 strongly **(J2)**. Olig2-lineage astrocytes were visualized by their GFP immunoreactivity **(J3)**, which co-localized with GAT-3. Panel **(J4)** is the merged image of **(J1–J3). (K)** An Olig2-lineage astrocyte in the cerebral cortex also expresses GAT-3. PV, parvalbumin; CPu; caudate-putamen; GP, globus pallidus. Scale bar: **(J)** 200 μm; **(K)** 20 μm.

During lineage tracing studies using adult Olig2-GFP mice (Tatsumi et al., 2008; Islam et al., 2009; Okuda et al., 2009), we noticed that GFP-labeled mature astrocytes cluster in specific brain nuclei such as the GP (Figures 1, 2; also see the first section of the “Results”). The finding prompted us to examine the effects of physical exercise on those astrocytes in the present study.

**Figure 3:**
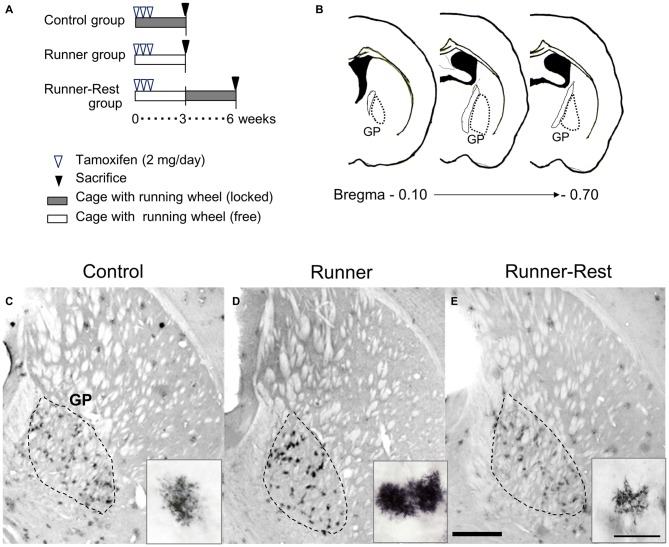
**GFP immunoreactivities of Olig2-lineage cells in the GP correlate with overall running activities. (A)** Schematic representation of experimental schedules. Double transgenic mice (12 weeks old) were divided into three groups (*n* = 6 per group). Every mouse received intraperitoneal tamoxifen injections for the first 3 days (2 mg/day). Control and Runner groups were given locked and free running wheels, respectively, for 3 weeks. The Runner-Rest group mice ran freely for 3 weeks and were then placed in cages with locked wheels for another 3 weeks. **(B)** Histological localization of the GP of the mouse (Franklin and Paxinos, 1997). The numerical values indicate posterior distances (mm) from the plate of bregma. **(C–E)** Each group was sacrificed at the end of the period and brain sections were subjected to immunohistochemical detection of GFP. The area demarcated with a dotted line represents the GP. GFP immunoreactivity was visualized with the DAB chromogenic reaction. Insets show representative GFP-positive cells (Olig2-lineage). Note that GFP-immunoreactive cells in the Control group **(C)** are less conspicuous than those in the Runner group **(D)** due to their weak immunoreactivity. Also note that cells in the Runner-Rest group (**E**; inset) show simpler morphology than those (**D**; inset) in the Runner group. Scale bar in **(E)** is also for **(C,D)** and indicates 1 mm. Scale bar in the inset of **(E)** is also for those in **(C,D)** and indicates 30 μm.

Figure 3A depicts the experimental schedule of the present study. Tamoxifen (TM, Sigma Aldrich, Japan) treatment methods have been described previously (Mori et al., 2006). To label Olig2 promoter-active cells, we intraperitoneally injected 2 mg of TM, dissolved in corn oil at a concentration of 20 mg/ml, into Olig2-GFP mice (12 weeks old) once a day for the first 3 days (Figure 3A). The TM treatment *per se* was harmful to neither neurons nor glia in the GP, judged from the absence of morphological signs of degeneration, apoptosis and necrosis (data not shown).

Mice were divided into three groups: animals in the first group were kept in a standard cage with a locked running wheel to prevent wheel running (Control group); the second group of mice were given an unlocked running wheel for voluntary free running (Runner group) for 3 weeks; and mice in the third group were kept for an additional 3 weeks with the running wheel locked after the 3-week running period (Runner-Rest group). To confirm wheel-running activity, the number of rotations was monitored and transmitted to a computer (wireless running wheel system; Med Associates, USA) continuously during the experiment. The average total running distances converted from rotation numbers during the 3 weeks were approximately 100 km (ca. 5 km/day). Unbiased comparison of the GFP-labeled cell morphologies among the three groups relies on the stability of GFP expression for three or 6 weeks. The reporter line (ROSA26-GAP43-EGFP) has been employed in lineage tracing studies for long periods (Dimou et al., 2008; Guo et al., 2009, 2012; Chung et al., 2013) with stable expression. We also confirmed that the GFP expression continued for up to 1 year after TM treatment (data not shown). In the present study, 18 Olig2-GFP mice were used for morphological analyses: nine mice for immunofluorescence analyses and nine mice for immunoelectron microscopic study, comprising three mice each in the Control, Runner and Runner-Rest groups.

### Tissue Preparation and Immunohistochemistry

After 3 weeks (Control and Runner groups) or 6 weeks (Runner-Rest group), mice were perfused with 4% paraformaldehyde (PFA) in phosphate-buffered saline (PBS). Brains were removed and post-fixed in 4% PFA. Coronal sections were cut at 40-μm thickness on a vibratome (Dosaka EM, Japan).

Immunohistochemistry was performed as described previously (Tatsumi et al., 2008) The primary antibodies were used at the following dilutions: anti-GFP (1:10,000, rabbit polyclonal, A6455, Thermo Fisher Scientific; 1:2000, rat monoclonal IgG2a, clone GF090R, Nacalai Tesque, Japan), anti-NG2 proteoglycan (1:200, rabbit polyclonal, AB5320, EMD Millipore, USA), anti-adenomatous polyposis coli protein (APC) (1:500, mouse monoclonal, clone CC-1, EMD Millipore), anti- S100 calcium-binding protein B(S100β) (1:5000, rabbit polyclonal, AB41548, Abcam, Japan), anti-GABA transporter 3 (1:200, rabbit polyclonal, AB1574, EMD Millipore). Alexa conjugated antibodies (1:1000, Jackson ImmunoResearch Laboratories, UK), or biotinylated goat anti-rabbit IgG (1:200; Vector Laboratories, USA) were used as secondary antibodies.

To minimize artifactual variation in fluorescence intensity across animals and groups, GFP immunofluorescence procedures for all sections of three groups were performed simultaneously under the same staining conditions.

### Immunoelectron Microscopy

All procedures were carried out as described previously (Tatsumi et al., 2008). After immunoreaction with DAB containing 0.03% H_2_O_2_ solution, sections were treated with 1% osmium tetroxide, dehydrated through a graded ethanol series, and flat-embedded in epoxy resin (Epon812 resin embedding kit, TAAB Laboratories Equipment, Japan). The resin was polymerized for 2 days at 60°C. We then carefully dissected out well-isolated GFP-positive cells in the GP from a flat-embedded section under a light microscope. Dissected GFP-positive cells were then sectioned at 80-nm thickness on an ultramicrotome (Leica EM UC7, Leica Microsystems, Japan) and ultra-thin sections were collected on formvar-coated single-slit grids. The sections were observed under a JEOL transmission electron microscope (TEM; JEM-1400plus, JEOL, Japan) without any counterstaining.

### Histological Quantification

All image analyses were performed by an investigator who was blind to the experimental groups to avoid subjective bias. We focused on the GP by restricting brain sections of pre-set stereotaxic coordinates (−0.10 mm to −0.70 mm posterior from the bregma, see Figure 3B; Franklin and Paxinos, 1997).

### Densitometric Analyses of Confocal Laser Microscopic Data

To analyze semi-quantitatively the complexity of astrocytic arborization, we initially tried to apply Scholl analysis, which is widely employed in quantification of cellular process arborization. However, Scholl analysis was not suitable for analyzing the “bushy” astrocytic processes, because the fine processes were too complex and too densely packed to count at the light microscopic level (representative images are shown in Figures 1D1, 2H1). We reasoned that fine and complex astrocytic processes should have more plasma membrane than simple ones; membrane-targeted GFP should then reflect the amount of plasma membrane, on the assumption that GFP molecules are evenly distributed on cellular membranes. We therefore measured the mean optical density (OD) of GFP immunofluorescence of an individual cell and correlated the OD value with the morphological complexity of that cell.

**Figure 4:**
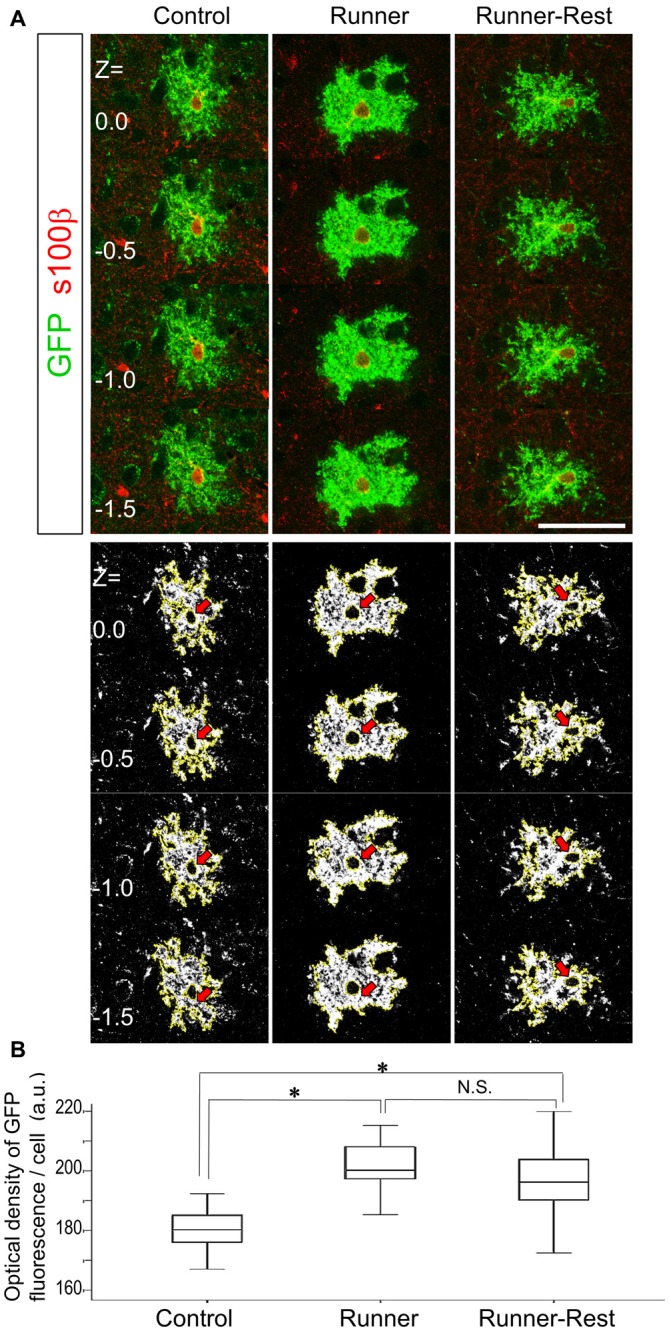
**Optical densities of GFP-immunoreactive cells correlate with overall running activities. (A)** Upper panels show representative optical sections of GFP (Alexa488; green fluorescence) and S100β (Alexa594; red fluorescence) double-positive cells in the three experimental groups visualized by confocal laser scanning microscopy (0.5 μm intervals between each optical section). Note that S100β primarily labels astrocytic nuclei. To avoid measurement bias, optical sections were confined to those displaying S100β nuclear labeling. Lower panels show actual ImageJ-processed images with yellow outlines and correspond to upper panels. Red arrows indicate subtracted nuclear regions. Scale bar: 30 μm (for all panels). **(B)** Optical densities of GFP-positive cells in the Control, Runner and Runner-Rest groups (30 cells from three animals in each group). The Runner group exhibited significantly higher mean optical densities than those of the Control group. The Runner-Rest group also displayed a significant difference from the Control group. The mean optical densities of the Runner-Rest group tended to decrease but were not significantly different from those of the Runner group. One-way analysis of variance (ANOVA) with *post hoc* Tukey HSD test. Data are presented as Mean ± SEM. *p < 0.05.

For each GFP and S100β double-immunoreactive cell, we acquired *z*-stack multi-channel images at 0.5-μm intervals using a confocal laser scanning microscope (Nikon C2-NiE, Tokyo, Japan) with a 6× water-immersion objective lens (numerical aperture 1.45) at optical zoom of 1.5 (x-axis × y-axis, 1024 × 1024 pixels). We selected four adjacent images with S100β-positive nuclei (see Figure 4A, upper panels) from the *z*-stack images (total image depth: 1.5 μm) and subjected them to OD analyses (see below). Immunostaining with S100β enabled us to set scanning positions of a cell and to confirm the cellular identity of mature astrocytes, but not of other Olig2-lineage cells. To avoid measurement bias, all stack images were acquired using the same settings for pinhole, frame size, scan directionality, scan speed and image average. Green channel images were then converted to 8-bit grayscale images using software linked to the confocal laser scanning microscope. Mean OD values of grayscale images were determined using ImageJ Software (NIH, version 1.48) with the following procedures and parameters. The backgrounds of all images were fitted by “Subtract background” (rolling ball radius 50), and binary images were then created with “Auto threshold” (Otsu method; Otsu, 1979).

The borders of the regions of interest (ROI, cell area) were outlined with “Wand tool” (Regacy mode). The lower panels of Figure 4A show actual ImageJ-processed images with yellow outlines and subtracted nuclear regions. These images correspond to those in the upper panels (Figure 4A). The mean OD value of the outlined image was then calculated with ImageJ Software. The OD values of four *z*-stack images were averaged to acquire each cellular OD value. For quantitative comparison of morphological complexities of Olig2-lineage astrocytes among three groups, 30 GFP-positive cells from three animals per group were subjected to the OD analyses.

### Morphometry of Transmission Electron Microscopic Data

In addition to the light-microscopic OD analyses (see above), we evaluated how complex astrocytic processes were in the neuropil using immunoelectron microscopic images. Supplementary Figure S1A depicts how a field to be analyzed was set by sectioning an astrocyte and was composed from smaller fields. First, we identified the nucleus of a GFP-immunoreactive cell under the TEM and positioned it in the center area of an observation field at 3000× magnification. We then acquired a wide field image (30 μm × 30 μm) composed of nine compartments (10 μm × 10 μm) using the montage function of a digital camera fully integrated into the TEM operation system (JEOL). Various shapes of GFP-immunoreactive “fragments” were detected in the field of an ultra-thin section (Supplementary Figures S1B1,B2). We treated all fragments as parts of a single astrocyte, having dissected individual GFP-immunoreactive cells that were well isolated from others (see above); and it is well known that each astrocyte occupies a proprietary territory that barely overlaps with adjacent astrocytes (Halassa et al., 2007). The field size (30 μm × 30 μm) was equivalent to the territory of a single astrocyte, because we observed that the average diameter of Olig2-lineage astrocytes was about 30 μm (Figures 1–3).

We next traced and pseudo-colored the GFP-immunoreactive fragments (red color in Supplementary Figures S1C1,C2) using Paint software (Microsoft, WA, USA). This procedure was applied to eight compartments excluding the center one, which was the perinuclear region (Supplementary Figure S1C1). The reason we excluded the perinuclear region was that it is primarily occupied by the nucleus, cytoplasm and roots of primary processes of astrocytes, all of which hardly participate in structural plastic changes (Bushong et al., 2002). Finally, we extracted the pseudo-colored fragments (Supplementary Figure S1C2), counted them (Supplementary Figure S1D2) and calculated the total area they occupied in an astrocytic territory. These analyses were performed by following the commands of ImageJ Software: brightness/contrast and threshold values of images were adjusted to eliminate all background but highlight all pseudo-colored fragments (Supplementary Figures S1D1,D2). All fragments were extracted with “Wand tool” (Regacy mode), which rendered a yellow outline to each fragment (Supplementary Figure S1D2). We chose 20 GFP-positive astrocytes from three animals (6~7 cells/animal) in each experimental group, and the fragment numbers and total areas were subjected to statistical analyses.

We consider that these fragment analyses in a two-dimensional image reflect the three-dimensional complexity of astrocytic fine processes; the finer and more complex the astrocytic processes became, the more frequently the GFP-positive fragments occurred.

### Statistics

All statistical analyses were performed using IBM Statistical Package for Social Science (SPSS v23). Group differences were analyzed using one-way analysis of variance (ANOVA) followed by the Tukey HSD *post hoc* test. All data are expressed as means ± SEM. The significance level was set at *p* < 0.05.

## Results

### Olig2-lineage Astrocytes Cluster in the Normal Globus Pallidus

Olig2-lineage cells with bushy processes were detected by GFP immunohistochemistry in Olig2-GFP mice (Figure 1A). When we observed sections at low magnification, we noticed that the Olig2-lineage cells were not distributed uniformly, but clustered in specific brain nuclei such as the GP (Figures 1A–C). These cells expressed S100β, a mature astrocyte marker (Figures 1D, 2H). Although a majority of Olig2-lineage cells in the adult brain gave rise to oligodendrocyte-lineage cells, which expressed NG2-proteoglycan (oligodendrocyte precursor cell marker; Figure 2E) or APC (mature oligodendrocyte marker; Figures 2F,G), these type of cells were undetectable at this low magnification. Quantitative analyses of Olig2-lineage cells in the GP revealed that 87% of GFP cells were bushy-type (S100β positive), while the cerebral cortex contained 12% of them (Figure 2I). We examined whether Olig2-lineage astrocytes expressed GABA transporter-3 (GAT-3) by double-labeling immunohistochemistry. Consistent with previous reports (Galvan et al., 2010; Jin et al., 2011), GAT-3 was expressed strongly in the GP (Figure 2J), and Olig2-lineage bushy cell expressed GAT-3 (Figure 1E), suggesting that Olig2-lineage astrocytes are involved in GABA transmission. Consistent with this notion, it should be noted that Olig2-lineage astrocytes in the cerebral cortex also expressed GAT-3 (Figure 2K).

Immunoelectron microscopy also revealed that Olig2-lineage astrocyte with bushy morphology were protoplasmic astrocytes: cellular processes were extended into the neuropil (Figure 1F, arrows), their fine processes enwrapped or were closely apposed to the synapses (Figure 1G, arrows; arrowheads indicate synapses), and formed a perivascular endfoot (Figure 1H, arrows).

Taken together, these results indicated that Olig2-lineage mature astrocytes are relatively abundant in the normal adult GP, and prompted us to investigate their role(s) in motor functions.

### Changes in Olig2-lineage Astrocyte Morphology Correlate with Overall Running Activity

The detailed morphology of Olig2-lineage cells in the three experimental groups was visualized by GFP immunohistochemistry at three or 6 weeks after tamoxifen administration. Consistent with the immunolabeling data (Figures 1A, 2B), Olig2-lineage cells with bushy morphology were clustered in the GP (Figures 3C–E) in coronal sections. The bushy morphology became pronounced in cells of the Runner group relative to those of the Control group (Figures 3C,D, insets). Interestingly, the bushy morphology of the astrocytes seen in the Runner group at 3 weeks after TM treatment (Figure 3D, inset) appeared to revert to simple morphology after an additional 3-week sedentary period in the Runner-Rest group (Figure 3E, inset). Although we could not track the morphology of an individual cell in these running-rest paradigms, our data suggest that arborization of astrocytic processes became complex after wheel running and then simplified after the 3-week sedentary period.

To analyze morphological changes semi-quantitatively, we measured the OD of GFP that accumulated at the plasma membrane of astrocytic processes and compared the data among the Control, Runner and Runner-Rest groups. Assuming that the promoter activity of the ROSA locus is stable, the amount of GFP protein should correlate with the complexity of the plasma membrane of an astrocyte. Upper panels of Figure 4A show representative optical sections of a bushy astrocyte from each group with anti-nuclear S100β stain (Alexa594; red fluorescence). Lower panels show the corresponding ImageJ-processed images of the upper ones (nuclei are subtracted from further analyses; red arrows). These images were subjected to OD measurement using ImageJ Software. The cellular OD values in the Runner group were significantly higher than those of the Control group (Figure 4B; Control group = 180.4 ± 7.6; Runner group = 201.0 ± 8.4, arbitrary units (a.u.), *p* < 0.05, 30 cells from three animals in each group). The cellular ODs of the Runner-Rest group tended to decrease but their values were not significantly different from those of the Runner group (Runner-Rest group = 195.7 ± 12.3 (a.u.), 30 cells from three animals). There was also a significant difference between the Control and Runner-Rest groups. These results suggested that the morphological complexity of the Olig2-lineage astrocytes changed in accordance with overall running activities.

**Figure 5:**
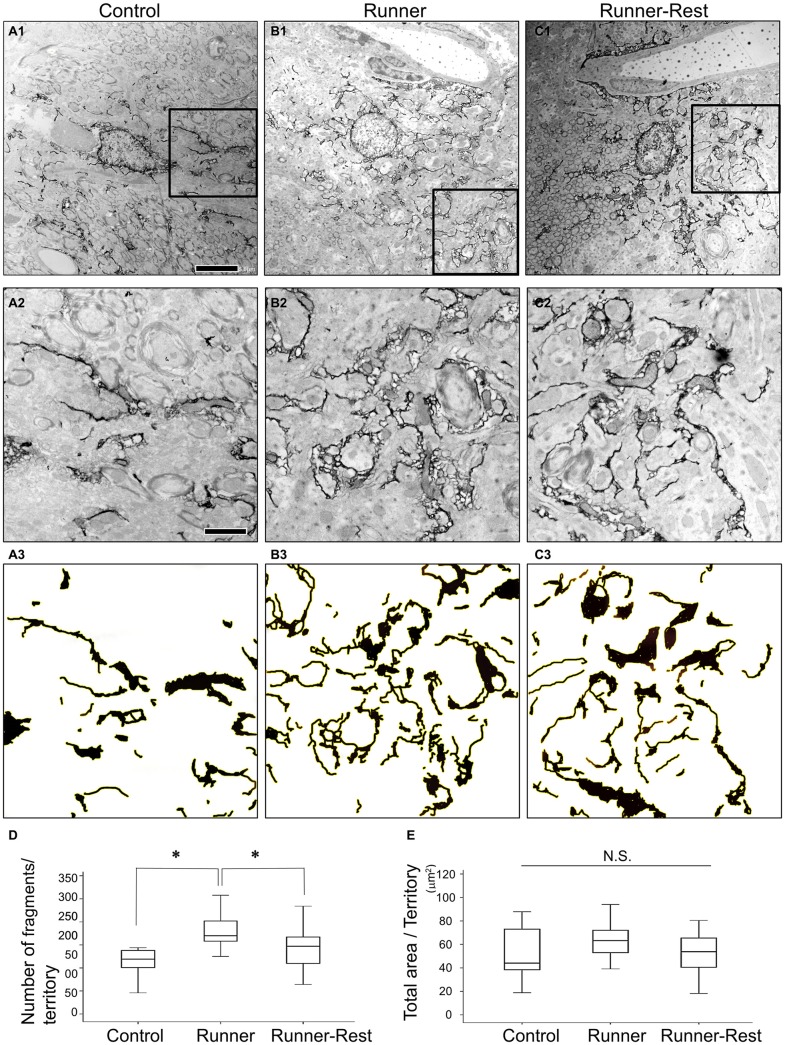
**(A1–C1)** Representative immunoelectron microscopic images of Olig2-lineage astrocytes in the Control **(A1)**, Runner **(B1)**, and Runner-Rest **(C1)** groups. These images are of single astrocytic territory (30 μm × 30 μm). **(A2–C2)** Squared areas (10 μm × 10 μm) in the panel **(A1–C1)** are shown in higher magnification, respectively. **(A3–C3)** Images of **(A2–C2)** were processed with ImageJ Software as done in the Supplementary Figure S1D2 for the Control **(A3)**, Runner **(B3)**, and Runner-Rest **(C3)** groups. Scale bar in **(A1)** is also for **(B1,C1)** and indicates 5 μm. Scale bar in **(A2)** is also for **(B2,C2,A3–C3)**, indicating 2 μm. **(D,E)** Quantitative data of morphometric analyses of GFP-immunoreactive fine processes (20 cells from three animals in each group). The number of fragments per territory was significantly higher in the Runner group than in the Control group, and that of the Runner-Rest group was significantly lower than the Runner group **(D)**. In contrast, there was no significant difference among total extracted areas of the three groups **(E)**. Statistical analyses were performed with one-way ANOVA with *post hoc* Tukey HSD test. Data are presented as Mean ± SEM. *p < 0.05.

Next, to further delineate the anatomical basis of the augmented astrocytic arborization, GFP-immunoreactive structures (i.e., astrocytic fine processes) were analyzed by immunoelectron microscopy. Since the average diameter of Olig2-lineage astrocytes in the GP was approximately 30 μm (Figures 1D,E, 3C–E), an area of 30 × 30 μm with the astrocytic nucleus in the center was defined for arborization analyses as a single astrocytic territory. We regarded individual pseudo-colored GFP-immunoreactive structures (red color in Supplementary Figures S1C1,C2) as “fragments” and extracted them with ImageJ (Supplementary Figure S1D2) to count their numbers and to summate the colored area in an astrocytic territory. Figures 5A–C shows representative immunoelectron microscopic images (30 μm × 30 μm; each cellular territory) of Olig2-lineage astrocytes in the Control, Runner, and Runner-Rest groups, respectively. Squared areas (10 μm × 10 μm) in the Figures 5A–C were magnified in Figures 5A–C and corresponding images processed with ImageJ Software of panels A2–C2 were shown in Figures 5A–C (for details of image processing procedures and morphometric analyses, please refer to Figure S1 in Supplementary Material and “Materials and Methods” Section). The fragment numbers per territory were significantly higher in the Runner group than in the Control group (Figure 5D; Control group = 0.15 ± 0.02; Runner group = 0.27 ± 0.02, 20 cells from three animals in each group), and those of the Runner-Rest group were significantly lower than those of the Runner group (Figure 5D; Runner-Rest group = 0.21 ± 0.01, 20 cells from three animals). In contrast, there was no statistically significant difference among total areas of the three groups (Figure 5E). These results indicated that Olig2-lineage astrocytes in the GP of Runner group mice had more ramified processes than those of the other two groups.

Together, these morphometric analyses at the ultrastructural level indicated that Olig2-lineage astrocytes in the GP can reversibly change their fine processes in their territory in response to overall running activities.

## Discussion

Physical exercise is regarded as being highly important in maintaining healthy functions of the brain, but the underlying mechanisms are still open to question (Hillman et al., 2008). The present study focused on the morphological changes of astrocytes with special reference to the following two aspects: first, we observed Olig2-lineage astrocytes rather than GFAP-positive astrocytes to examine the effects of physical exercise; and second, we concentrated on the GP, which exerts pivotal functions in motor regulation but has not previously been explored in terms of glial biology.

Bayraktar et al. (2015) reviewed recent work reporting that astrocytes are not homogeneous, but constitute heterogeneous populations that differ in their morphology, gene expression and function. Astrocytes are also allocated to spatially distinct regional domains during development (Molofsky et al., 2014). Regional heterogeneity may help to coordinate postnatal neural circuit formation and thereby to regulate eventual neuronal activity (Khakh and Sofroniew, 2015). In the present study, we focused on Olig2-lineage astrocytes, which may not constitute a major population of astrocytes but rather are localized to certain regions of the gray matter including the GP. Their localization patterns imply a correlation with local neural function. After a 3-week running period, Olig2-lineage astrocytes changed their morphology but their numbers seldom increased, because they are post-mitotic cells and are not generated from adult OPCs/NG2 glia (mitotic cells) in normal adult brain (Dimou et al., 2008; Guo et al., 2011). In other words, voluntary exercise did not induce proliferation of Olig2-lineage astrocytes, but stimulated the arborization of their fine processes. These astrocytes are different from so-called reactive astrocytes, which are characterized by hypertrophy of GFAP-containing main processes (Wilhelmsson et al., 2006), since GFAP expression by Olig2-lineage astrocytes in the GP was continuously weak during our running-rest paradigms. In addition, we did not observe an increase of the GFP-labeled area in the Runner group in the morphometric analyses (Figure 5E).

We focused on Olig2-lineage astrocytes localized in the GP, which is a component of the indirect pathway of the basal ganglionic circuits. The major input to the GP is GABAergic axons from the striatum, and GP neurons are themselves GABAergic (Chuhma et al., 2011), making inhibitory transmission a primary event (Jin et al., 2014). A traditional view of the basal ganglionic circuits is that the direct pathway accelerates body movements and the indirect pathway inhibits motor functions (Albin et al., 1989). This “accelerator-brake” model has recently been revisited and modified by new findings as follows: both pathways are simultaneously active (not active in turn), and coordinated outputs through the thalamic nuclei determine the activities of the motor cortex (Sano et al., 2013; Calabresi et al., 2014). Indeed, we found that Runner group mice in the present experimental paradigm expressed the immediate-early genes c-Fos, Arc and Zif268 in their GP neurons (Supplementary Figure S2).

In our experiments, voluntary exercise for 3 weeks induced astrocytic morphological change, suggesting a response to motor activities. Supporting our hypothesis, three sedentary weeks after the voluntary exercise period rendered astrocytic morphology simpler in the immunoelectron microscopic analyses (Figure 5). On the other hand, the OD analysis of confocal laser microscopic data failed to show a statistically significant difference between the Runner and Runner-Rest groups. The discrepancy between the two kinds of morphometric analyses may be attributed to their different detection sensitivities (Heller and Rusakov, 2015): membrane-targeted GFP labeled not only the plasma membrane but also mitochondrial (Figure 1H, asterisk) and endoplasmic reticulum membranes within the fine processes, suggesting that immunofluorescence analyses are less accurate than electron microscopic analyses in the morphometry of cellular shapes. We should take this point into account as a caveat in future morphometric studies, since immunofluorescence is much easier and less tedious to perform than immunoelectron microscopy.

The morphological plasticity observed after three sedentary weeks is unlikely to result from unhealthy conditions leading to cell death, because we never observed signs of cell death (i.e., clumped chromatin, discontinuous nuclear and plasma membranes, amorphous organelles or apoptotic bodies) during electron microscopic examination. In the electron microscopic analyses, we observed GFP-positive perisynaptic processes that were closely apposed to synapses (Figure 1G). In the past two decades, accumulating evidence has demonstrated that perisynaptic astrocytic processes (PAPs) are dynamic elements involved in reciprocal communication between astrocytes and neurons (Volterra and Meldolesi, 2005; Halassa et al., 2007; Allen and Barres, 2009; Gruber, 2009; Perea et al., 2009; Eroglu and Barres, 2010; Dityatev and Rusakov, 2011; Lavialle et al., 2011; Patrushev et al., 2013; Bernardinelli et al., 2014). This communication takes place at a “tripartite synapse” consisting of pre- and postsynaptic neurons and PAPs. PAPs are minute and fine astrocytic processes and are thought to regulate synaptic transmission by removing excess neurotransmitters and releasing gliotransmitters into synaptic clefts (e.g., ATP, glutamate, GABA, D-serine, and prostaglandin-E2; Volterra and Meldolesi, 2005; Gordon et al., 2009; Welberg, 2009; Hamilton and Attwell, 2010; Clasadonte et al., 2011; Jo et al., 2014). Through these mechanisms, astrocytes have been proposed to play a critical role in modulating synaptic transmission (Fellin et al., 2004), plasticity properties (Jourdain et al., 2007) and long-term potentiation (LTP; Henneberger et al., 2010; Rosenberg et al., 2013). For example, excitatory synapses in the hippocampus show activity-dependent remodeling and stabilization in learning and memory paradigms. Along with the remodeling of synapses, PAPs extend into the neuropil and enwrap neuronal terminals or closely appose synapses. Such astrocytic structural plasticity may contribute to learning and memory processes (Malinow and Malenka, 2002).

We observed that Olig2-lineage astrocytes in the GP express a GABA transporter, GAT-3 (Figures 1E, 2J; Galvan et al., 2010; Jin et al., 2011). GAT-3 is primarily localized to PAPs and controls inhibitory synaptic transmission by regulating GABA concentration in synaptic clefts (Minelli et al., 1996; Jin et al., 2011; Melone et al., 2014). A recent report demonstrated that astrocytic fine processes display actin-dependent motility, and this motility is triggered by intracellular Ca^2+^ elevation in response to neuronal activity in the hippocampus (Bernardinelli et al., 2014). However, it remains unclear what kind of molecular mechanism governs the motility and thereby induces morphological changes of astrocytes in the hippocampus (Bernardinelli et al., 2014). In the GP, some yet-unknown mechanisms must also underlie astrocytic morphological plasticity, and uncovering such mechanisms will be an important step toward understanding how the indirect pathway of the basal ganglia regulates motor functions.

The phenomenon of Olig2-lineage astrocytes reversibly transforming their fine processes in response to overall neuronal activity may occur in brain regions other than the GP, since we found that Olig2-lineage astrocytes distributed widely but unevenly in the mouse central nervous system (CNS; Figures 1A, 2B). An intriguing hypothesis is that Olig2-lineage astrocytes may be specifically subsidiary to inhibitory GABAergic transmission; this hypothesis is currently under investigation. We also do not yet know whether astrocytes of other lineages also change their morphology in response to neuronal activity. This question awaits further studies with different lineage-tracing strategies.

## Author Contributions

KT designed research; KT, HO, SM-T, AI, TT, TS and YT performed research; KT and AW analyzed data and wrote the article.

## Funding

This work was supported by JSPS KAKENHI Grant Numbers 15K06743 (Scientific Research (C)), 15K14354 (Exploratory Research), 26830029 (Young Scientists (B)) and 26293039 (Scientific Research (B)), and by the Takeda Science Foundation (JJ12010029).

## Conflict of Interest Statement

The authors declare that the research was conducted in the absence of any commercial or financial relationships that could be construed as a potential conflict of interest.
