# Supplementary material for: Voluntary Exercise Induces Astrocytic Structural Plasticity in the Globus Pallidus
Source: Front Cell Neurosci. 2016 Jun 21;10:165. doi: 10.3389/fncel.2016.00165 (PMC4914586; doi:10.3389/fncel.2016.00165)
Supplement: Supplementary file 1 [file Image_1.pdf]

Supplementary Figure-1

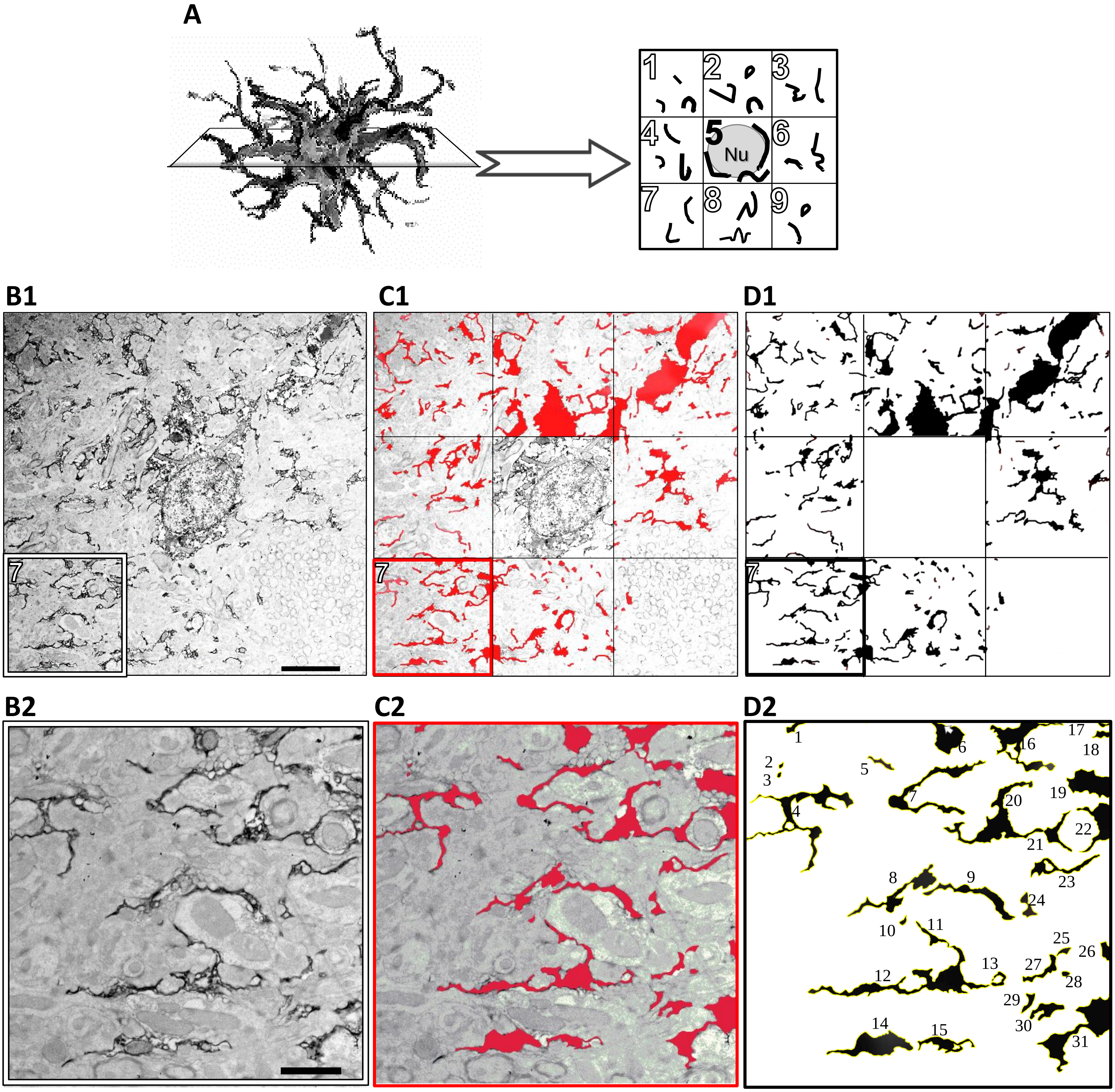

Supplementary Figure S1.

Morphometric analyses of immunoelectron microscopy of astrocytic fine processes.

(A) Schematic representation of how we set a field to be analyzed in an ultrathin section. The astrocytic processes appeared in a section as GFP-positive fragments with various shapes. The nucleus of GFP-positive cells was positioned to the center area of the view at x 3000 magnification, and the field was photographed using the montage function (3 x 3). A wide field image (30 μm x 30 μm) composed of nine fields (numbered 1 to 9; 10 μm x 10 μm) were regarded as a single astrocyte territory.

(B-D) Representative electron microscopic montage images subjected to morphometric analyses of GFP-immunoreactive fine processes (**B1**) and pseudo-colored GFP-immunoreactive fragments except for the center area (**C1**). The pseudo-colored fragments were extracted with ImageJ software (**D1**). B2, C2 and D2 are high-magnification views of the #7 field in B1, C1 and D1, respectively. D2 indicates an actual analysis screen of ImageJ software. In this field, 31 fragments are extracted with yellow outlines and numbered. Scale bar: **B1** 5 μm; **B2** 2 μm.

Supplementary Figure-2

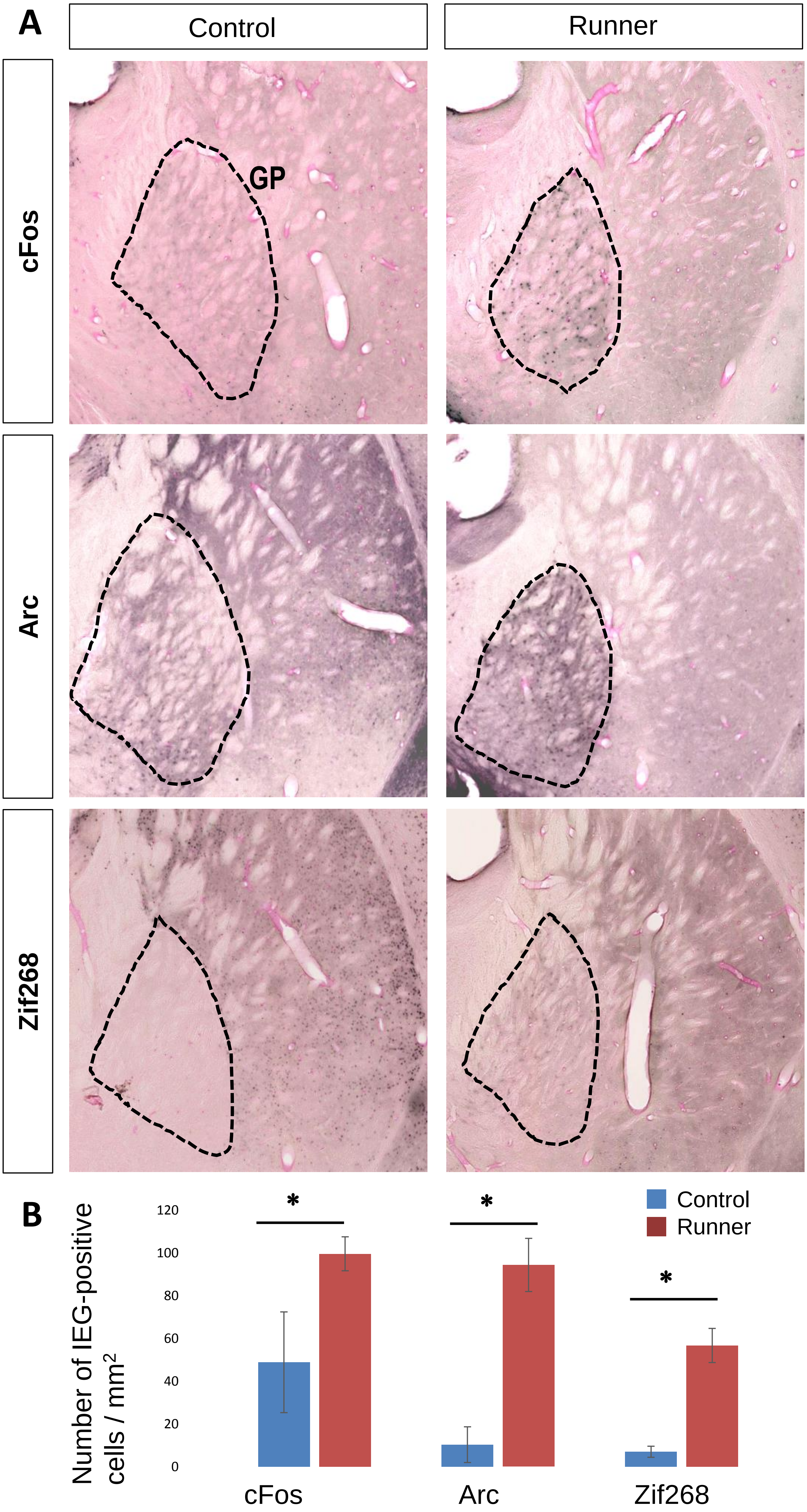

Supplementary Figure S2:

The protein products of immediate-early genes (IEGs) in the globus pallidus of runner and control mice.

(**A**) Representative coronal sections immunostained for IEG proteins (DAB reactions in neuronal nuclei) with a light eosin counterstaining. Expression of cFos, Arc and Zif268 proteins increased markedly in the GP of the Runner group mice as compared to those of the Control group mice. All animals were sacrificed at 9 AM (1 hour after the end of dark period) to ensure that voluntary exercise effects were manifested in IEG expressions. (**B**) Quantitative analysis of IEG protein expression in the GP. The numbers of IEG protein-positive nuclei were significantly increased in accordance with running activities. Statistical analyses were performed with one-way ANOVA with post-hoc Tukey HSD test. Data are presented as means ±S.E.M. $^*p<0.05$.
